# L-carnitine protects the lung from radiation-induced damage in rats via the AMPK/SIRT1/TGF-1ß pathway

**DOI:** 10.1007/s00210-024-03157-w

**Published:** 2024-05-22

**Authors:** Nasıf Fatih Karakuyu, Alper Özseven, Süleyman Emre Akın, Hasan Ekrem Çamaş, Özlem Özmen, Çağla Cengiz

**Affiliations:** 1 Department of Pharmacology, Faculty of Pharmacy, Suleyman Demirel University, Isparta, Turkey; 2 Department of Radiation Oncology, Faculty of Medicine, Suleyman Demirel University, Isparta, Turkey; 3 Department of Thoracic Surgery, Faculty of Medicine, Suleyman Demirel University, Isparta, Turkey; 4 Department of Pathology, Faculty of Veterinary Medicine, Burdur Mehmet Akif Ersoy University, Burdur, Turkey; 5 Undergraduate Student, Faculty of Medicine, Suleyman Demirel University, Isparta, Turkey

**Keywords:** L-carnitine, Lung, Radiation, Radiotherapy, Rat

## Abstract

Radiotherapy (RAD) is a common cancer treatment method, but it can have unintended lung side effects. L-carnitine (LCAR) is an amino acid with antioxidant and anti-inflammatory properties. This study aims to demonstrate the effects of LCAR against radiation-induced acute lung injury and to elucidate its possible protective molecular mechanisms. A total of 32 Wistar albino rats were separated into four groups: control, RAD (10 Gy once on 1st day), RAD + LCAR (intraperitoneally, 200 mg/kg/d, for 10 days), and LCAR. At the end of the experiment, the rats were euthanized, and the lung tissues were collected for histopathological, immunohistochemical, biochemical, and genetic analysis. Emphysema, pronounced hyperemia, increased total oxidant status, and increased caspase-3 and TNF-α immunostainings were all seen in the lung tissues of the RAD group. LCAR treatment reduced these negative effects. In addition, AMPK and SIRT1 gene expressions increased in the RAD + LCAR group compared to the RAD group, while TGF-1ß gene expression decreased. While RAD caused major damage to the lungs of rats, LCAR application reduced this damage through antioxidant, anti-inflammatory, and anti-apoptotic mechanisms. Specifically, LCAR reduced fibrosis while attenuating RAD-induced inflammation and oxidative stress via the AMPK/SIRT1/TGF-1ß pathway. Therefore, LCAR can be considered a supplement to reduce complications associated with RAD.

**Graphical abstract:**

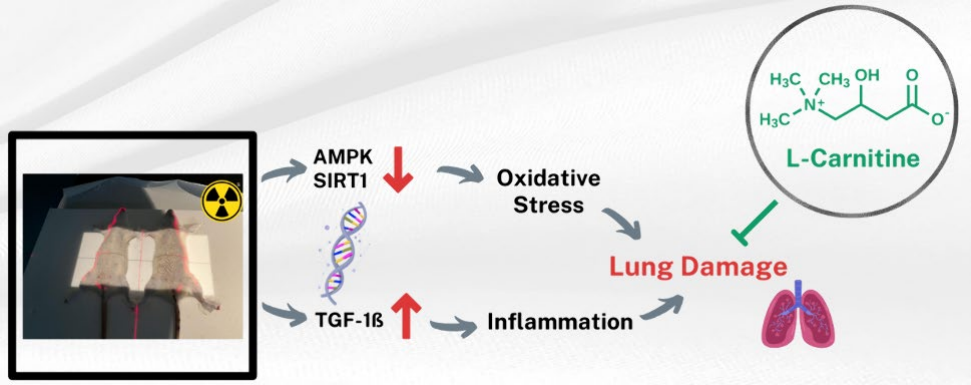

## Introduction

It is known that cancer treatment has three important pillars that have been practiced for years: surgery, chemotherapy, and radiotherapy. The mentioned treatment methods can be applied in combination, considering parameters such as the type of cancer, whether it is benign or malignant, or whether it metastasizes or not.

While every treatment has benefits, it may have unintended side effects (Barazzuol et al. 2020). These treatment methods, especially radiotherapy, have many harms. Ionizing radiation can cause damage by physically affecting the structural parts of the cell (membrane, cytoskeleton, DNA and RNA structures, proteins), as well as causing oxidative damage by creating many free oxygen radicals at the cell level. Cellular damage secondary to radiotherapy is especially important in lung tissue due to its high blood supply (Ping et al. 2020).

Reactive oxygen species (ROS) and proinflammatory cytokines caused by radiotherapy as well as cytotoxic agents are necessary for the physiological function of cells, but excessive production of these molecules damages cellular components such as proteins, lipids, and DNA (Park et al. 2022). The production of ROS and inflammatory cytokines such as tumor necrosis factor-alpha (TNF-α), transforming growth factor 1 beta (TGF-1β), and interleukin-6 (IL-6) results in inflammation which causes tissue damage and directly induces DNA damage (Damm et al. 2022). This has encouraged many researchers to investigate the protective effects of some potent ROS-scavenging antioxidants on radiation-induced apoptosis and normal tissue damage. Many in vivo and in vitro studies are designed for this purpose. Animal studies, in particular, have an important place in this regard (Sinha et al. 2013).

L-carnitine (LCAR) is an amino acid derived from lysine and methionine in the liver and kidneys. It is stored in muscles, the heart, brain, and sperm cells. It is obtained mostly through a diet of meat and other animal foods. One of the most important tasks that LCAR performs in the body is transporting fats, especially long-chain fatty acids, to the cell mitochondria. Here, oxidized fatty acids produce ATP and are used as energy. It has properties that increase fat burning in metabolism, strengthen muscles, and increase sperm motility (Kurt and El 2011). It also has antioxidant activity. A study reported that LCAR showed anti-inflammatory and antioxidant properties through the adenosine monophosphate-activated protein kinase (AMPK)/ serine/threonine kinase (AKT)/ nuclear factor-κB (NF-κβ) signaling pathway in rats’ brain tissue (Salama and Elgohary 2021).

This study aims to demonstrate the effects of LCAR against radiation-induced acute lung injury in rats and to elucidate its possible protective molecular mechanisms.

## Materials and methods

### Ethical approval

This study, which the Ethics Committee approved with the decision of the Suleyman Demirel University Animal Experiments Local Ethics Committee dated 26.01.2023 and numbered 01/115, was carried out in the laboratories of the Suleyman Demirel University Experimental Animals and Medical Research Application and Research Center.

### Experimental design

The experiment involved the use of Wistar albino rats weighing between 250 and 300 g. They were kept in a controlled environment with a room temperature maintained at 21–22 °C, a 12-h light/12-h dark cycle, and humidity kept at 60 ± 5%. The rats were cared for in accordance with standard procedures for feeding and housing, and were provided with sufficient care throughout the study.

Thirty-two rats were randomly divided into four groups with eight rats in each group were formed as follows:

- Group I (Control): To create equal stress with the radiation process, the rats were anesthetized on the first day and no procedures were performed. Then, 1 ml of saline was given intraperitoneally (i.p.) every day for 10 days.
- Group II (RAD): A single dose of 10 Gy X-ray at a dose rate of 0.6 Gy/min was applied to the whole body to the anesthetized rats, and 1 ml of saline was administered i.p. daily for 10 days (Aşcı Çelik and Toğay 2023).
- Group III (RAD + LCAR): A single dose of 10 Gy X-ray at a dose rate of 0.6 Gy/min was applied to the whole body to the anesthetized rats, and 200 mg/kg LCAR was administered i.p. daily for 10 days (Abd Eldaim et al. 2018).
- Group IV (LCAR): To create equal stress with the radiation process, the rats were anesthetized on the first day and no procedures were performed. Then, 200 mg/kg LCAR was administered i.p. every day for 10 days.

### Irradiation procedure

**Fig. 1:**
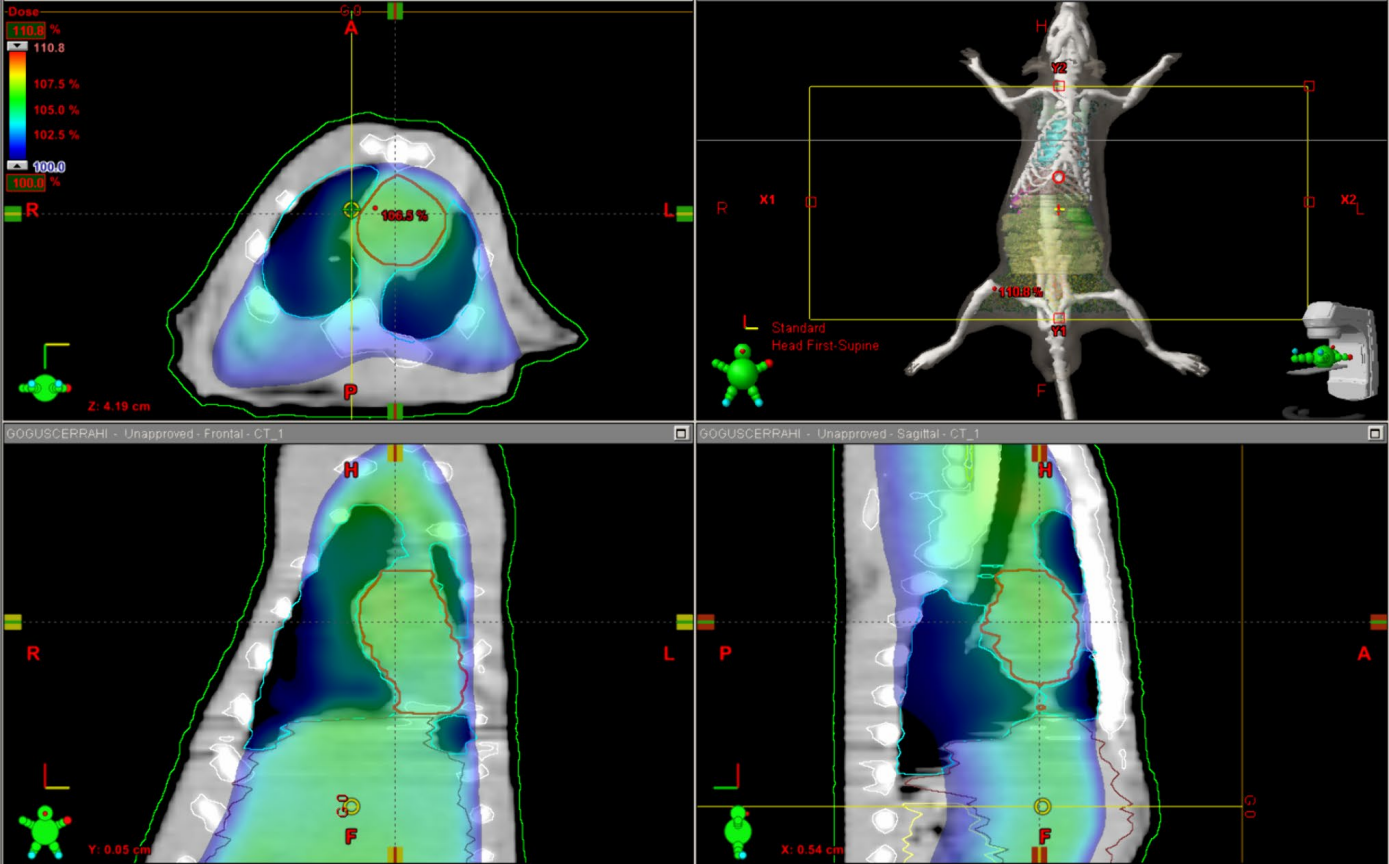
Irradiation planning process on rats

Radiotherapy treatment plans were created using the Eclipse treatment planning system on the Varian DHX linear accelerator (Varian Medical Systems, Palo Alto, CA, USA) with a fixed source-to-surface distance of 98.5 cm. A single field with a size of 14 cm × 30 cm was created that covers from the chest down to the tail of a rat. A 6 MV x-ray beam and an Anisotropic Analytical Algorithm dose calculation algorithm were used in the planning process, and a 10 Gy was prescribed for irradiation (Fig. 1). The temperature in the room during the irradiation was 22 °C.

### Euthanasia methodology

Twenty-four hours after the last drug administration, rats were euthanized under ketamine (90 mg/kg)/xylazine (8–10 mg/kg) anesthesia by taking a high volume of blood from the vena cava inferior, and the lung tissues were separated after euthanasia. Half of the taken tissues were placed in a 10% formaldehyde solution for histopathological and immunohistochemical analyses, while the remaining tissues were wrapped in aluminum foils and stored at −80 °C for genetic and biochemical analyses.

### Histopathological evaluation

All of the rats were humanely killed at the end of the investigation. Lung tissue samples were taken during the necropsy and put in a 10% buffered formalin solution for storage. After a 2-day fixation, tissue samples were routinely processed using a fully automated tissue processor (Leica ASP300S; Leica Microsystem, Nussloch, Germany) and embedded in paraffin wax. Five micrometer sections were cut using a fully automated Leica RM 2155 rotary microtome (Leica Microsystem, Nussloch, Germany) from the paraffin blocks. The sections were stained with hematoxylin-eosin (HE), coverslipped, and examined with a light microscope. According to the degree of hyperemia, edema, inflammatory cell infiltrations, and epithelial cell loss, the histopathological lesions were graded from 0 to 3. The scoring system is shown in Table 1. The histological scoring criteria have been modified from the study by Passmore et al. (2018).

**Table 1:** Histological scoring system for the evaluation of lung inflammation

Score	Mean
0	No damage
1	Slight hyperemia, interstitial edema, increased alveolar thickness, peribronchial infiltration, alveolar epithelial loss, and scantly interstitial inflammatory cell infiltration
2	Moderate hyperemia, alveolar edema, increased alveolar thickness, peribronchial infiltration, alveolar and bronchial epithelial loss, and mild interstitial inflammatory cell infiltration
3	Severe hyperemia, alveolar edema, marked increased alveolar thickness, peribronchial infiltration, alveolar, bronchiolar, and bronchial epithelial loss, and marked interstitial inflammatory cell infiltration

### Immunohistochemical examination

Two series lung tissue of sections taken from each paraffin block and drawn on poly-L-lysine coated slides were stained using recombinant anti-caspase-3 antibody [EPR18297] (ab184787) and recombinant anti-TNF alpha antibody [RM1005] (ab307164) for the expression of TNF-α and caspase-3 (cas-3) in accordance with the manufacturer’s instructions. The concentration of each primary antibody is 1/100. Using streptavidin-alkaline phosphatase conjugate and a biotinylated secondary antibody, immunohistochemistry was carried out on the sections after the primary antibodies had been incubated for an overnight period. We used the Mouse and Rabbit Specific HRP/AEC (ABC) Detection IHC Kit (ab93705) as a secondary antibody. Aminoethyl carbazole (AEC) was the chromogen used. All of the primary and secondary antibodies were purchased from Abcam (Cambridge, UK). Antigen dilution solution was used as negative control rather than primary antibodies. A specialized pathologist from another university conducted each evaluation on blinded samples.

At an objective magnification of ×40, the percentage of cells that were immunostained positively for each marker in ten different areas on each slide for all groups was determined. Using the ImageJ program (National Institutes of Health, Bethesda, MD, version 1.48), the output of the image analyzer was tallied. Before counting, the images were cropped and divided into color channels, and any artifacts were removed. The software’s counting tool was used to count the cells after first selecting the cells inside the regions of interest with a selection tool. Only cells with a marked red stain were considered positive, and the red hue was used to determine positive staining. The Database Manual Cell Sens Life Science Imaging Software System (Olympus Co., Tokyo, Japan) was used to take microphotos.

### Biochemical analysis

Rat lung tissues weighing about 200 mg each were homogenized using an ULTRA-TURRAX T-25 homogenizer (Janke & Kunkel, IKA® Werke, Germany) using phosphate-buffered saline at a ratio of 1:9 (10 mM Na_2_HPO_4_, 1.8 mM KH_2_PO_4_, 2.7 mM KCl, 137 mM NaCl, pH 7.4). Homogenized lung tissues were then centrifuged for 10 min at 10,000 rpm. Total antioxidant status (TAS [mmol Trolox Eq/g protein]) and total oxidant status (TOS [µmol H_2_O_2_/g protein]) levels were measured using Erel’s methods using a biochemistry autoanalyzer (Beckman Coulter AU 5800, USA) with the supernatants obtained after centrifugation (Erel 2004, 2005). Oxidative stress indexes (OSI) were calculated by the formula (TOS/TAS)*100.

### Reverse transcription-polymerase chain reaction (RT-qPCR)

Using the manufacturer’s protocol, RNA was isolated from homogenized tissues with the GeneAll RiboEx (TM) RNA Isolation Kit (GeneAll Biotechnology, Seoul, Korea). The amount and purity of the RNAs obtained were measured with the BioSpec-nano nanodrop (Shimadzu Ltd. Kyoto, Japan) device. One microgram RNA was used for cDNA synthesis. For cDNA synthesis, A.B.T. ™ cDNA Synthesis Kit (Atlas Biotechnology, Turkey) was carried out in a thermal cycler according to the protocol. Primer designs were made by detecting specific mRNA sequences and testing possible primer sequences using the NCBI website. The sequences of the primer sequences used are shown in Table 2. Expression levels of genes were measured in a Biorad CFX96 (CA, USA) real-time PCR instrument using 2X SYBR Master Mix (Nepenthe/Turkey). In the study, the ACTB gene was used as a housekeeping gene. The reaction mixture was prepared according to the manufacturer’s protocol to a final volume of 20 µl. The resulting reaction mixture was placed in a real-time qPCR device with thermal cycling determined according to the kit manufacturer’s protocol, and each sample was studied in 3 replications. PCR conditions, initial denaturation 95 °C 10 min 1 cycle, denaturation 94 °C 15 s, and annealing/extension 55 °C 30 s were applied as 40 cycles. Relative mRNA levels were calculated by applying the 2^- ΔΔCt^ formula to the normalized results.

**Table 2:** Primary sequences, product size, and accession numbers of genes

Genes	Primary sequence	Product size	Accession number
ACTB (HouseKeeping)	F: CCCGCGAGTACAACCTTCTT	481 bp	NM_031144.3
	R: AACACAGCCTGGATGGCTAC		
AMPK	F: TCGGCAAAGTGAAGATTGGAG	308 bp	NM_023991.1
	R: GTAGTCCACGGCAGACAGAA		
SIRT1	F: GGTAGTTCCTCGGTGTCCT	152 bp	NM_001414959.1
	R: ACCCAATAACAATGAGGAGGTC		
TGFB1	F: CCAGATCCTGTCCAAACTAA	218 bp	NM_021578.2
	R: TTTTGTCATAGATTGCGTTG		

*F* forward, *R* reverse, *ACTB* actin B, *AMPK* AMP-activated catalytic subunit alpha 2, *SIRT1* sirtuin 1, *TGFB1* transforming growth factor beta 1

### Statistical analysis

SPSS 19.0 package program and one-way ANOVA Tukey test were used to statistically analyze, and the significance level was accepted as *p* < 0.05.

## Results

### Histopathological findings

**Fig. 2:**
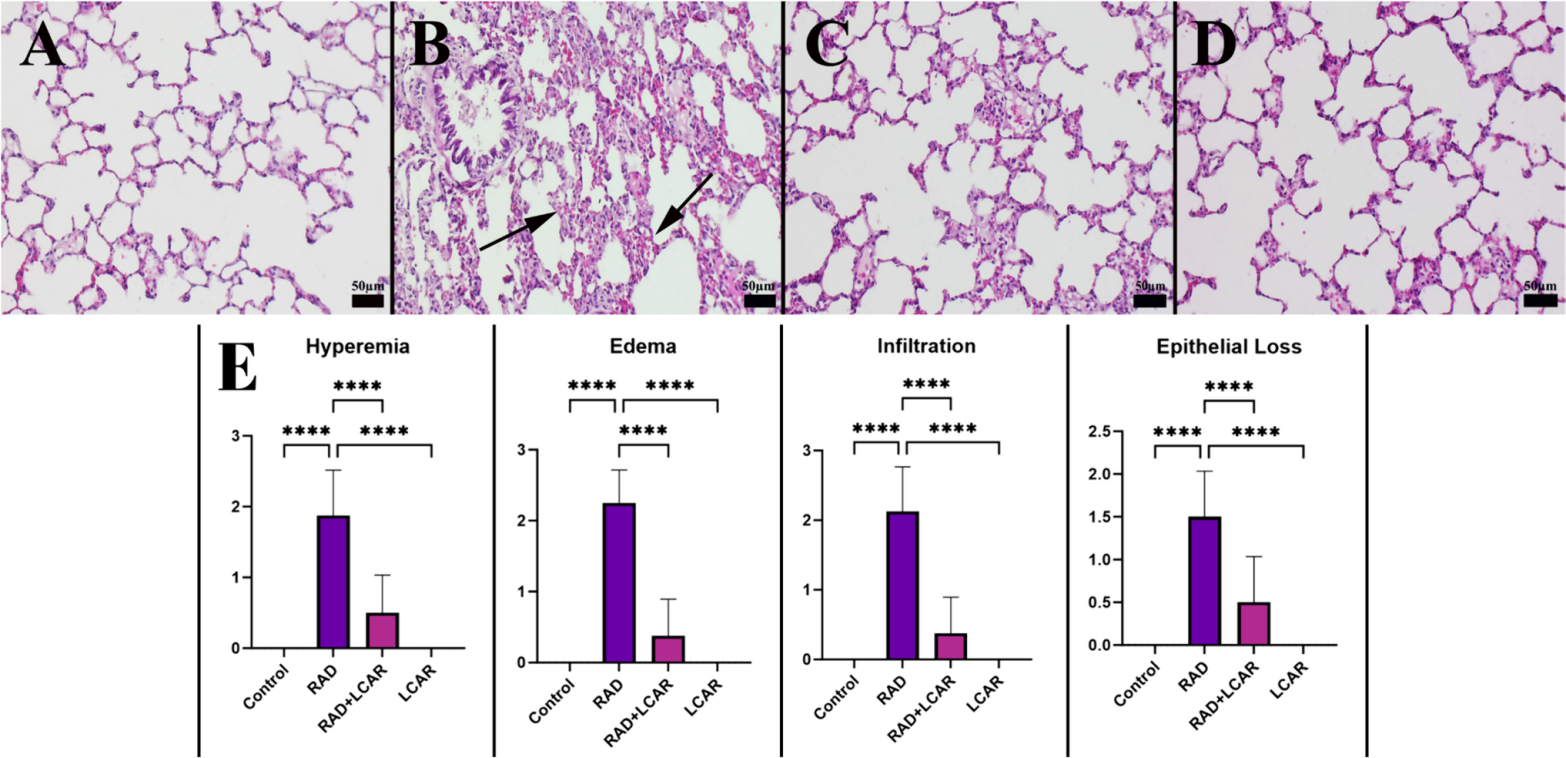
Histopathological appearance of lungs and statistical analysis of histopathological evaluation scores between the groups. **A** Normal lung histology in the control group. **B** Increased septal tissue thickness (arrows) and inflammatory reaction in lungs in the RAD group. **C** Decreased pathological findings in the RAD+LCAR group. **D** Normal tissue architecture in the LCAR group, HE, scale bars = 50 µm. **E** Statistical analysis of histopathological evaluation scores. Statistical analysis was performed with one-way ANOVA Tukey test “****” represents *p* ≤ 0.0001

The lungs from the control and LCAR groups showed normal tissue organization by histology. Emphysema, pronounced hyperemia, edema, increased septal tissue thickness, inflammatory cell infiltrations, and epithelial loss were all seen in the RAD group. LCAR treatment reduced the negative effects in the RAD+ICAR group (Fig. 2).

### Immunohistochemical findings

**Fig. 3:**
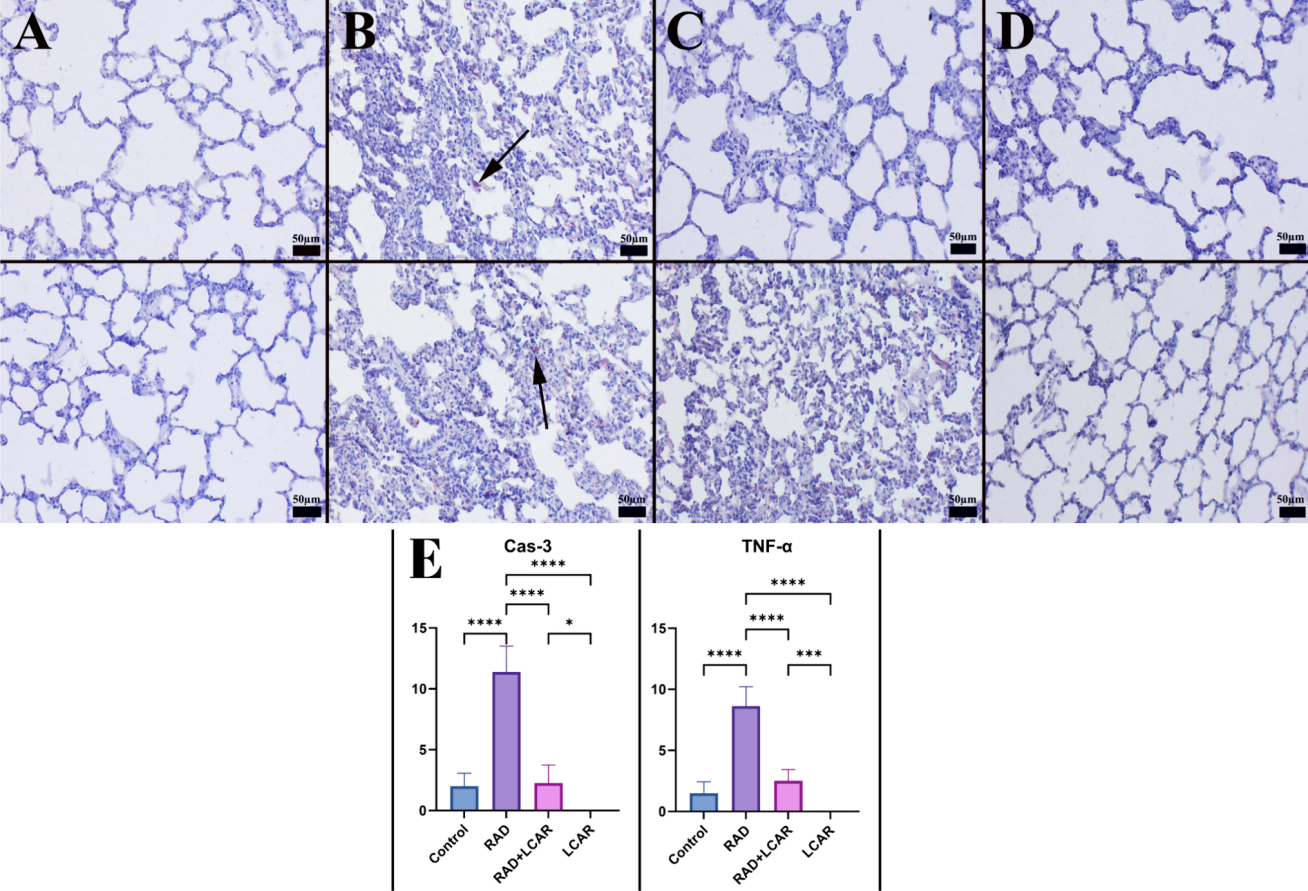
Caspase-3 (upper row) and TNF-α (below row) expressions and statistical analysis of immunohistochemistry expressions between the groups. **A** Negative expression in control group. **B** Increased expression (arrows) in RAD group. **C** Decrease expression in RAD+LCAR group. **D** No immunoreaction in LCAR group. Streptavidin biotin peroxidase method, scale bars = 50 µm. **E** Statistical analysis of immunohistochemistry expressions. Statistical analysis was performed with one-way ANOVA Tukey test “*” represents *p* < 0.05, “***” represents *p* < 0.0005, and “****” represents *p* ≤ 0.0001

The slides of immunohistochemical staining from the control group showed either no or very low Cas-3 and TNF-α expression. Expression of Cas-3 and TNF-α substantially increased in the RAD group. TNF-α and Cas-3 expression both increased after LCAR treatment. Using markers, the LCAR group had expressions that were comparable to those of the control group (Fig. 3). Expressions were frequently seen in alveolar epithelial cells, alveolar macrophages, and inflammatory cells.

### Biochemical analysis results

**Fig. 4:**
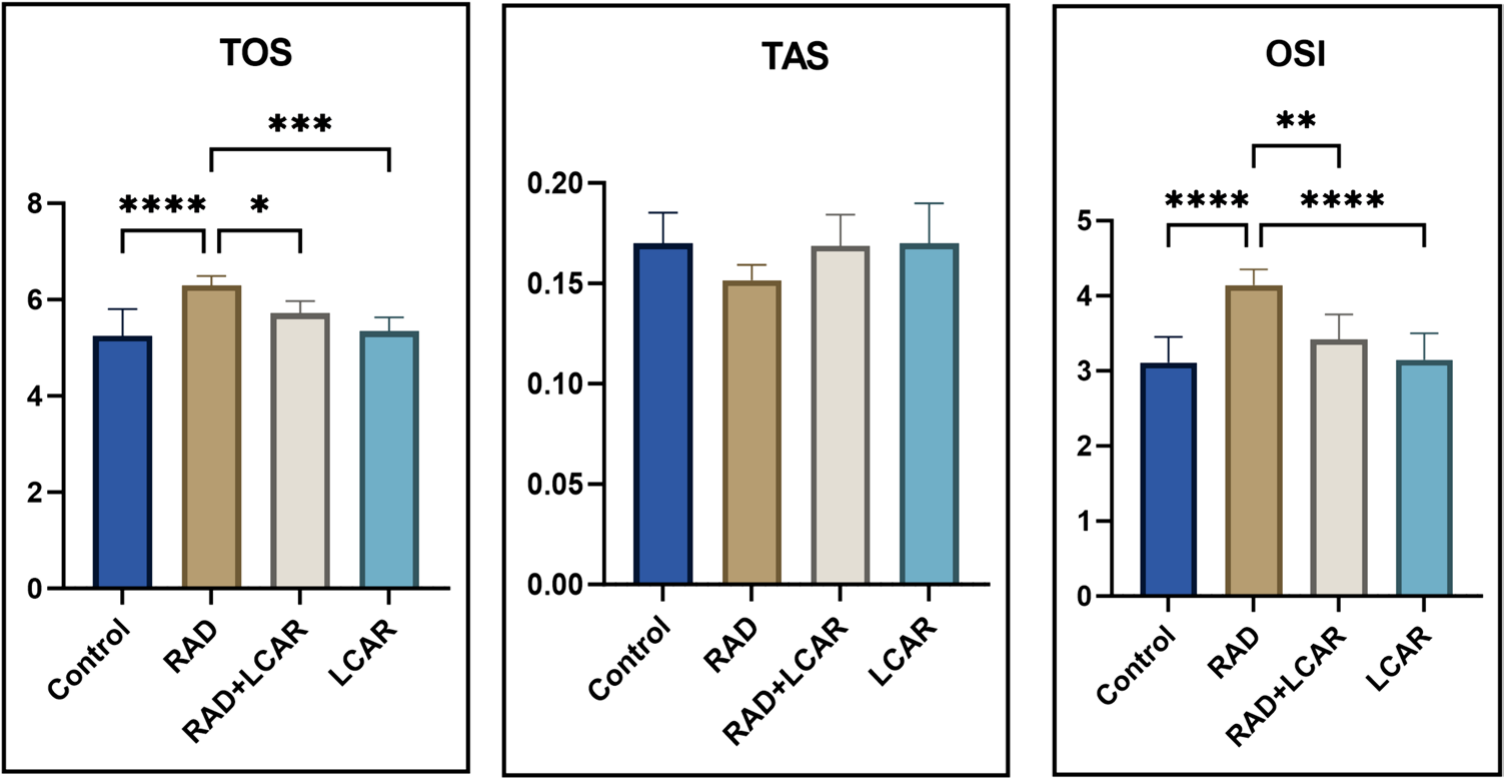
Biochemical analysis results. Statistical analysis of biochemical parameters was performed with one-way ANOVA Tukey test “*” represents *p* < 0.05, “**” represents *p* < 0.005, “***” represents *p* < 0.0005, and “****” represents *p* ≤ 0.0001

According to the results of the examinations performed on lung tissues, TOS and OSI values increased significantly in the RAD group compared to the control group (*p* ≤ 0.0001, for both). These values were found to be significantly lower in the RAD+LCAR (*p* < 0.05, *p* < 0.005, respectively) and LCAR (*p* < 0.05, *p* < 0.005, respectively) groups compared to the RAD group. TAS value was lower in the RAD group than in other groups, but it was not statistically significant (Fig. 4).

### RT-qPCR relative mRNA expression results

**Fig. 5:**
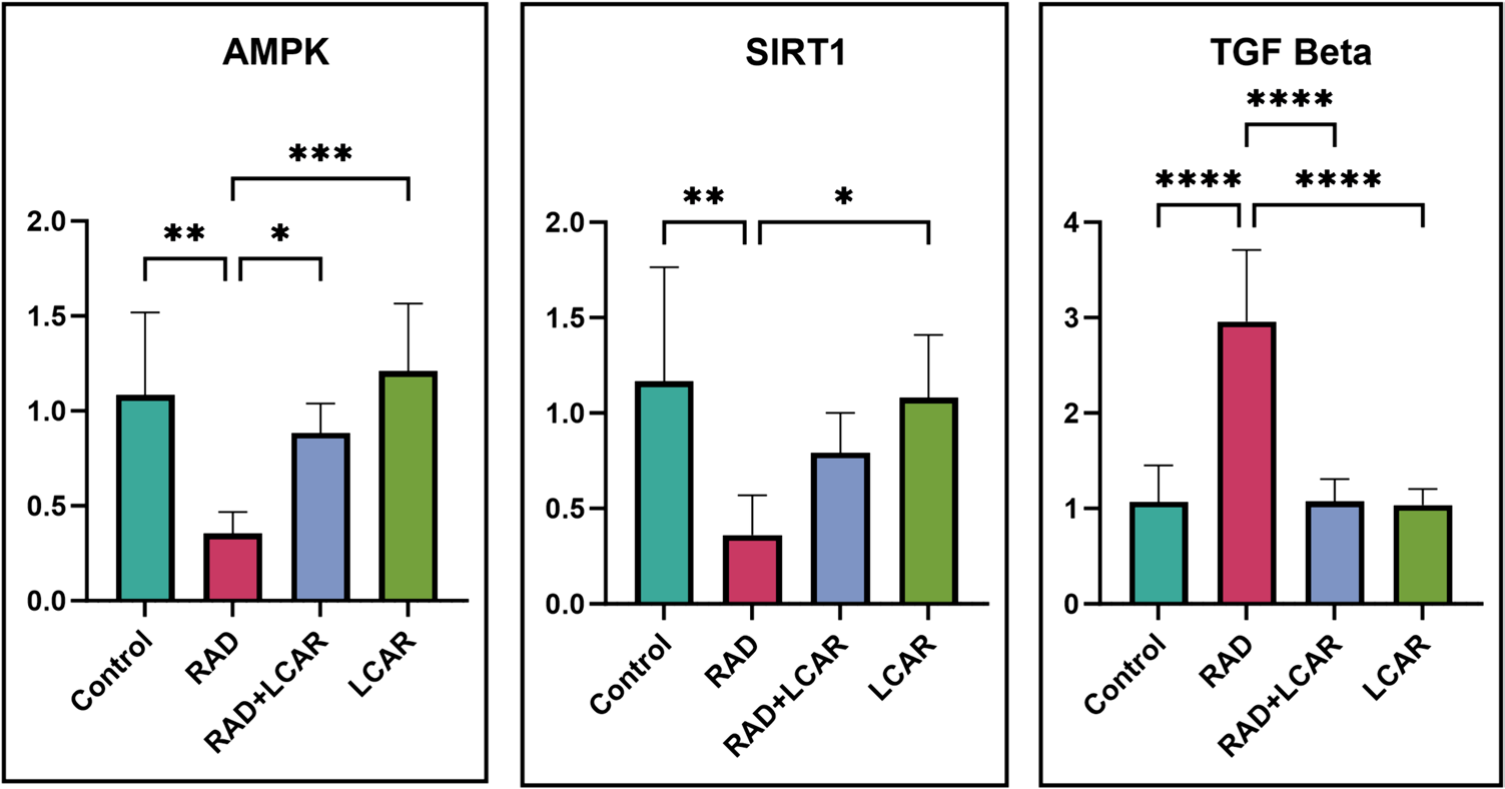
RT-qPCR relative mRNA expression results. Statistical analysis of relative mRNA expressions was performed with one-way ANOVA Tukey test “*” represents *p* < 0.05, “**” represents *p* < 0.005, “***” represents *p* < 0.0005, and “****” represents *p* ≤ 0.0001

When the mRNA expression results were examined, AMPK and SIRT1 expressions decreased significantly in the RAD group compared to the control group (*p* < 0.005, for both). This increase was reversed significantly in the RAD+LCAR group (*p* < 0.05) for AMPK. Likewise, a significant increase was observed for AMPK and SIRT1 expressions in the LCAR groups compared to the RAD group (*p* < 0.0005, *p* < 0.05, respectively). TGF Beta value was significantly higher in the RAD group compared to the control group (*p* ≤ 0.0001), and a significant decrease was observed in the RAD+LCAR and LCAR groups compared to the RAD group (*p* ≤ 0.0001, for both) (Fig. 5).

## Discussion

As it is known, radiotherapy may cause damage to different parts of the body. The lung is one of the organs where these damages are most common. Experimental studies show that radiation application causes histopathological changes in lung cells (Luo et al. 2023; Seyedpour et al. 2023). Radiation causes fibrosis, especially in lung epithelium, through damage mechanisms such as oxidative stress and inflammation (Li et al. 2024).

Previous studies have shown that radiotherapy can directly cause lung damage through inflammation in rats (Wan et al. 2022). Inflammatory findings such as hyperemia, edema, and epithelial loss observed in the RAD group in this study indicate that the applied radiation dose causes lung damage. In addition, the decrease in these pathological changes in the RAD+LCAR group shows that LCAR reduces lung damage at the cellular level. The immunohistochemical analysis displayed an increase in TNF-α values in the RAD groups of this study, indicating the initiation of inflammation. The decrease in these values seen in the RAD+LCAR groups indicates that inflammation decreases with the effect of LCAR. Like our results, Abdel-Emam et al.’s study on rats demonstrated the protective effect of LCAR on the liver through its anti-inflammatory properties (Abdel-Emam and Ali 2022).

Fibrosis can be directly induced by radiation, or it can appear as a more severe pathological condition in the presence of additional triggers such as inflammation. Changes in the expression of some genes in cell nuclei provide information about the possible mechanisms of drug action. TGF-1ß is a protein that plays a vital role in intercellular communication and regulates many cellular processes; its biological effect generally focuses on cellular signal transduction and tissue homeostasis (Zhang 2018). In vitro and in vivo studies have shown that radiation causes fibrosis in the lung by increasing the expression of the TGF-1ß gene (Zhou et al. 2022; Chen et al. 2024). In a study by Abd Eldaim et al., they found that the increase in liver TGF-1ß expressions caused by high-fat nutrition decreased with LCAR (Abd Eldaim et al. 2018). Similarly, our study observed a decrease in this gene expression in the RAD+LCAR group compared to the RAD group. This indicates that LCAR can reduce the pathological effects of radiation-causing fibrosis.

Inflammation can lead to an increase in the production of free radicals in cells, resulting in oxidative stress. Previous studies have shown that radiation causes oxidative stress in the lung tissue of rats (Doğru et al. 2024). In a study conducted on rats by Boyacioglu et al., they showed that contrast material-induced oxidative stress in the kidneys was reduced by LCAR (Boyacioglu et al. 2014). Parallelly, in the RAD+LCAR group of this study, the OSI value, which is one of the markers of oxidative stress, decreased significantly compared to the RAD group.

AMPK is a protein kinase complex that regulates cellular energy levels. When cell energy levels decrease or the cell is under stress (e.g., oxidative stress), AMPK levels decrease (Zannella et al. 2011; Mahmoud Moustafa et al. 2021). SIRT1 is a protein belonging to the sirtuin family and plays an important role in the regulation of cellular aging and metabolic homeostasis. It has been suggested that SIRT1 plays a role in mitochondria biogenesis, and in this way, oxidative stress can be reduced by improving mitochondrial function (Hsu et al. 2016). Additionally, SIRT1 is known to increase the expression of antioxidant enzymes (Emamgholipour et al. 2016). In short, activation of these genes may help cells cope with oxidative stress and reduce the negative effects caused by this condition. According to the results of this study, the administration of LCAR to rats treated with radiation reversed the decrease in the levels of these genes compared to rats treated with radiation alone. As a contribution to the existing literature, it can be concluded that LCAR exhibits antioxidant properties (Kaya et al. 2015; Sepand et al. 2016).

When cells are exposed to oxidative stress, reactive oxygen species can cause harm to the proteins present within the cell. Activation of critical apoptosis proteins such as Cas-3 can trigger cell death, a process known as apoptosis. Previous studies have shown that radiotherapy can cause activation of apoptosis in rat testicles by inducing Cas-3 (Ibrahim et al. 2019). In this study, the high Cas-3 immunostaining in the group that received only RAD application indicates that the cells were driven to apoptosis. The fact that this immunostaining was low in the groups administered LCAR is evidence of the antioxidant properties of the drug.

## Conclusion

In conclusion, while RAD caused major damage to the lungs of rats, LCAR application reduced this damage through antioxidant, anti-inflammatory, and anti-apoptotic mechanisms. Specifically, LCAR reduced fibrosis while attenuating RAD-induced inflammation and oxidative stress via the AMPK/SIRT1/TGF-1ß pathway. Therefore, if more detailed experimental and clinical studies support the results, LCAR can be considered a supplement to reduce RAD-related complications.

## Data Availability

No datasets were generated or analysed during the current study.
